# Identification of climate factors related to human infection with avian influenza A H7N9 and H5N1 viruses in China

**DOI:** 10.1038/srep18094

**Published:** 2015-12-11

**Authors:** Jing Li, Yuhan Rao, Qinglan Sun, Xiaoxu Wu, Jiao Jin, Yuhai Bi, Jin Chen, Fumin Lei, Qiyong Liu, Ziyuan Duan, Juncai Ma, George F. Gao, Di Liu, Wenjun Liu

**Affiliations:** 1 CAS Key Laboratory of Pathogenic Microbiology and Immunology, Institute of Microbiology, Chinese Academy of Sciences, Beijing, 100101, China; 2 Department of Geographical Sciences, University of Maryland, College Park, Maryland, 20740, USA; 3 State Key Laboratory of Earth Surface Process and Resources Ecology, Beijing Normal University, Beijing, 100875, China; 4 Network Information Center, Institute of Microbiology, Chinese Academy of Sciences, Beijing, 100101, China; 5 College of Global Change and Earth System Sciences, Beijing Normal University, Beijing, 100875, China; 6 School of Mathematical Sciences, Beijing Normal University, Beijing, 100875, China; 7 CAS Key Laboratory of Zoological Systematics and Evolution, Institute of Zoology, Chinese Academy of Sciences, Beijing, 100101, China; 8 State Key Laboratory for Infectious Disease Prevention and Control, National Institute for Communicable Disease Control and Prevention, Chinese Center for Disease Control and Prevention, Beijing, 102206, China; 9 Institute of Genetics and Developmental Biology, Chinese Academy of Sciences, Beijing 100101, China

## Abstract

Human influenza infections display a strongly seasonal pattern. However, whether H7N9 and H5N1 infections correlate with climate factors has not been examined. Here, we analyzed 350 cases of H7N9 infection and 47 cases of H5N1 infection. The spatial characteristics of these cases revealed that H5N1 infections mainly occurred in the South, Middle, and Northwest of China, while the occurrence of H7N9 was concentrated in coastal areas of East and South of China. Aside from spatial-temporal characteristics, the most adaptive meteorological conditions for the occurrence of human infections by these two viral subtypes were different. We found that H7N9 infections correlate with climate factors, especially temperature (TEM) and relative humidity (RHU), while H5N1 infections correlate with TEM and atmospheric pressure (PRS). Hence, we propose a risky window (TEM 4–14 °C and RHU 65–95%) for H7N9 infection and (TEM 2–22 °C and PRS 980-1025 kPa) for H5N1 infection. Our results represent the first step in determining the effects of climate factors on two different virus infections in China and provide warning guidelines for the future when provinces fall into the risky windows. These findings revealed integrated predictive meteorological factors rooted in statistic data that enable the establishment of preventive actions and precautionary measures against future outbreaks.

Avian influenza viruses (AIVs) are negative-sense, single-stranded, enveloped RNA viruses that belongs to the influenza A genus of the *Orthomyxoviridae* family. Human AIV infections have caused severe economic losses and public concern worldwide. The occurrences of most influenza virus infections in humans display strong seasonal patterns. For instance, in northern latitudes, influenza viruses circulate from November to March^1^. However, in tropical and subtropical regions, the seasonal characteristics are divergent, with some areas experiencing annual epidemics coinciding with the local rainy season^2^,^3^,^4^. The influence of physical environmental factors and anthropogenic environmental factors on the prevalence of AIVs has been analyzed in some studies^5^,^6^. In actuality, AIV outbreaks are affected by complex factors, including climate, topography, poultry density, and human population density^7^,^8^. According to current data, poultry outbreaks increase with increasing human population density, combined with close proximity to lakes or wetlands, increased temperatures, and reduced precipitation during the cold season of highly pathogenic avian influenza (HPAI) H5N1^9^.

Human avian influenza virus H5N1 and H7N9 infections cause severe disease, including fatal cases. Both of these viruses are of avian origin and were directly transmitted from avians to humans. Different from seasonal influenza viruses with high infectivity and widespread outbreak patterns, these two AIVs often cause sporadic and small outbreaks, despite the lack of human-to-human transmission ability, so other more complex reasons were involved. Because these two viruses are associated with both wild and domestic bird populations, it is likely that environmental factors play a significant role in the spread of the viruses. Generally, wild birds may act as the primary spreading agent for the outbreak of the influenza virus^10^. After the movement of the virus from wild birds to poultry, the virus may spread from flock to flock and farm to farm through several ways^11^. These include wild bird migration, poultry-wild bird interaction, and human proximity to poultry. For example, poultry trade areas that are in proximity to wild waterfowl habitats, such as wetlands or lakes, can facilitate virus exchange between wild birds and poultry^12^,^13^. Animal model data shows that the transmission of IAV is more likely at low temperatures and relative humidity conditions^14^. However, the prevention of secondary spread can be achieved through restricting bird movement and proper biosecurity procedures. Given the mobility of wild birds and the challenges of sustained control, it is of great importance to identify which environment factors correlate with the occurrence of these two viruses.

Both the H5N1 and H7N9 influenza viruses are of avian origin and have similar infectivity to humans, but their transmission region, outbreak timing, and host range are diverse. Outbreaks of H5N1 in wild birds are concentrated in the central part of Europe, while outbreaks in poultry are mainly found in Eastern Europe, with human infections mainly in Southeast Asia and Eastern Europe^9^. Human infections with H7N9 are found in China, corresponding to its high prevalence in certain LPMs (live poultry markets)^15^, while the occurrence of H7N9 in wild birds is rare^16^.

Previously, data have indicated that environmental factors affect the prevalence of H5N1 and H7N9, with the infection and spread of the two viruses being closely correlated with bird habitats, migration, and local climate factors (*e.g.*, temperature and relative humidity)^17^. However, we found that both viruses have species-specific manifestations; the outbreak patterns of these two viruses in humans are not coincident with infections in wild birds or waterfowl. The reasons for this discrepancy could be due to the prevalence of the viruses in wild birds and waterfowl, the proximity of humans to these animals, and/or characteristics of the viruses themselves. The susceptibility of humans to the viruses due to host immune function and inhibition of mucociliary clearance by the inhalation of cold, dry air could also be a factor.

In the present study, we compared the physical environmental factors and anthropogenic environmental factors that were present during H5N1 and H7N9 AIV outbreaks, as these viruses have similar endemic areas, host ranges, and are the main concerns to public health in recent years in China. Understanding the outbreak patterns of these viruses is important to identify high-risk populations and areas, as well as to establish preventive actions and precautionary measures against future outbreaks.

## Results

### Spatial characteristics of H7N9 cases and H5N1 cases

As the temperature varied heterogeneously in gradients in different provinces, we classified the affected provinces into the three commonly used geographical sectors that have similar meteorological characteristics: South (south area of the Yangtze River), Central (the Yellow River to the south and the Yangtze River to the north), and North China (north area of the Yellow River). South China is comprised of Guangxi, Guangdong, and Fujian provinces, Central China is comprised of Sichuan, Guizhou, Hubei, Hunan, Jiangxi, Anhui, Jiangsu, Shanghai, and Zhejiang provinces, and North China is comprised of Shaanxi, Shanxi, Henan, Hebei, Shandong, and Liaoning provinces, as well as Beijing and Tianjin (municipal cities). As shown in Table 1, the H5N1 influenza infection cases mainly occurred in South, Central, and Northwest China, with the most (eight cases) in Hunan Province. In contrast to H5N1, the occurrence of H7N9 in mainland China was concentrated in the coastal areas of South China.

### Climatic characteristics of H7N9 cases and H5N1 cases

**Figure 1:**
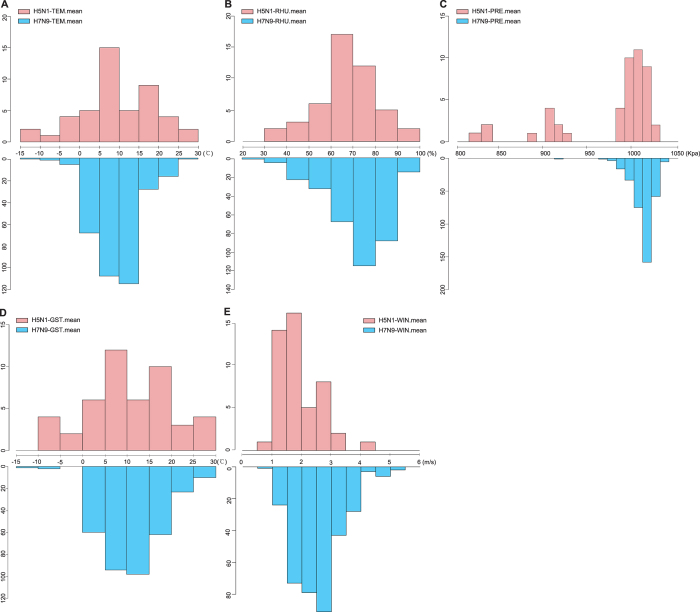
Temperature-Humidity distribution of H5N1 and H7N9 influenza infection. (**A**) Medial temperature (TEM) of H5N1 and H7N9 influenza infection, (**B**) medial humidity (RHU) of H5N1 and H7N9 influenza infection, (**C**) atmosphere pressure (PRS) of H5N1 and H7N9 influenza infection, (**D**) medial ground surface temperature (GST) of H5N1 and H7N9 infection, and (**E**) medial wind speed (WIN) of H5N1 and H7N9 infection. The vertical axis represents the number of cases.

To understand the discrepancies in climate factors between H7N9 and H5N1 infections, the null hypothesis was tested using the T-test. As shown in Table 2, the *p*-values for RHU.mean, PRS.mean, and WIN.mean were <0.05. These results imply that there were significant differences between H7N9 and H5N1 infections in terms of humidity, pressure, and wind speed. H5N1 infections occurred with the highest frequency in a humidity range of 60–70%, while H7N9 infections occurred in the 70–80% rage (Fig. 1B). In terms of atmospheric pressure, the cases of H5N1 were concentrated at 990–1020 kPa, while the occurrences of H7N9 infection were found at 1000–1030 kPa (Fig. 1C). In addition, as the composition of meteorological factors, the wind speed during H5N1 cases was concentrated at 1–2 m/s, while the occurrences of H7N9 infection were found at 2–3 m/s wind speed (Fig. 1F). Temperature and specific humidity were the best individual influence factors for influenza infection. According to null hypothesis 1, both the mean value of the air temperature and the mean value of the ground surface temperature displayed no significant difference comparing H5N1 and H7N9 infections. H5N1 influenza occurred with the highest frequency in a temperature range of 5–10 °C, with a second peak in the range of 15–20 °C. H7N9 influenza occurred with the highest frequency in a temperature range of 10–15 °C, with a second peak in the interval of 5–10 °C. Consistent with the TEM value, the H5N1 influenza infection cases were relatively more prevalent when the ground surface temperature was 5–10 °C, with a second peak at 15–20 °C; H7N9 influenza infection cases peaked at 10–15 °C and 5–10 °C, respectively (Fig. 1A,D).

### Correlation between climate factors and influenza infection

**Figure 2:**
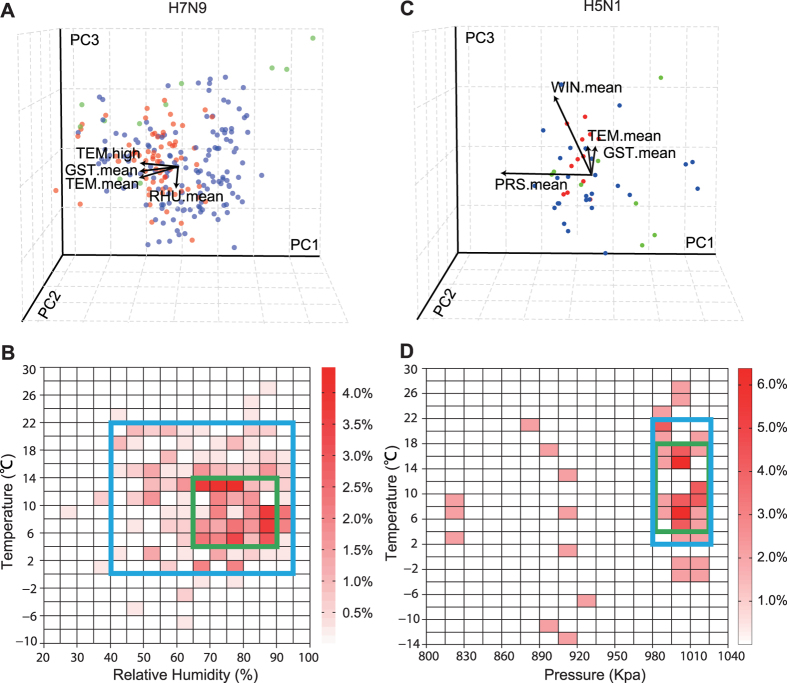
PCA and risk windows of H7N9 (**A**) and H5N1 (**C**) infection. PCA of the H7N9 infections with the climatic parameters. The first three principle components are used as axes. The green dots indicate cases in North China, while the blue and red dots indicate cases in Central and South China, respectively. Heat map of human infections against temperature and RHU for H7N9 infection (**B**) and TEM and PRS for H5N1 infection (**D**). Each cell represents the percentage of human infections. The green box represents a high-risk window, and the blue box represents a moderate-risk window.

To determine the correlation between climate factors and influenza infection, we established a null hypothesis that states that H5N1/H7N9 infection is independent of climate factors. The Chi-squared test was used to test this hypothesis. As shown in Table 3, we found that the *p*-value for all of climate is <0.05. These results demonstrate that there were significant relationships between climate factors and H5/H7 influenza infection. To further understand the relationships among these factors with H5/H7 infection, we conducted principal component analysis (PCA) for all climate factors employed in our study. As shown in Table 4, Fig. 2A and Supplementary Video S2, the results from the H7N9 influenza cases indicate that the first principle component, whose dominant contributors are temperature variables (*i.e*., TEM.mean, TEM.high, TEM.low, and GST.mean) explain 49.11% of the total variance; the second principle component, which is dominated by relative humidity variables (*i.e.*, RHU.mean and RHU.low), explains 24.20% of the total variance. The third principle component was dominated by wind speed (WIN.mean) and other variables and only explains 14.77% of the total variance. The total contribution of these three sets of climatic variables accounted for 88.04% of the total contribution.

Similar to the H7N9 influenza cases, the first H5N1 principle component was led by temperature variables (*i.e.*, TEM.mean, TEM.high, TEM.low, and GST.mean) and explains almost half of the total variance (46.92%, see Table 4, Fig. 2C and Supplementary Video S1). In contrast to H7N9, the second principle component of H5N1 was dominated by pressure (*i.e.*, PRS.mean), which explains 17.69% of the total variance, and the third principle component included humidity and other variables, explaining 16.17% of the total variance. The total contribution of these three sets of climatic variables accounted for 80.78% of the total contribution. From the above analysis, we can see that among all of the climatic variables, temperature and humidity were the key factors for H7N9 infection. For H5N1 infection, temperature and atmospheric pressure were the key factors, though humidity also had significant effects.

### Risk profile for H7N9 and H5N1 virus infection

**Figure 3:**
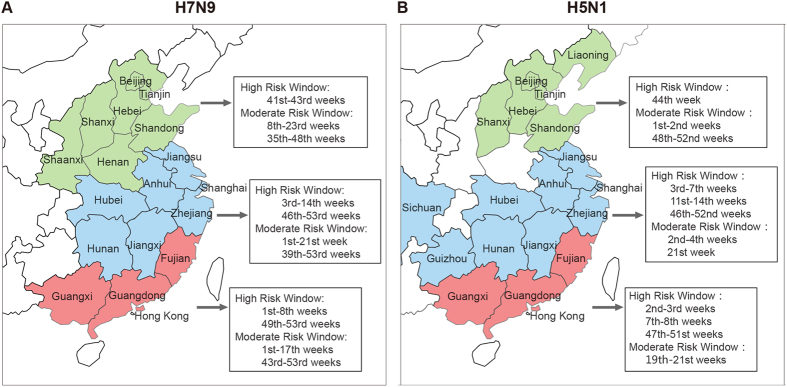
Predicted risk windows for possible human H7N9 (**A**) and H5N1 (**B**) infections in China. Predicted periods of climate conditions that are conducive for the spread of H7N9 based on the temperature and relative humidity ranges in North (green), Central (blue), and South (red) China. The indicated climate high- and moderate-risk windows indicate periods where vigilance for the control of H7N9 or H5N1 infection should be increased. Adobe Illustrator software was used for map depiction.

A heat map was generated to evaluate the factors conducive to the spread of H7N9 (Fig. 2B). Based on the heat map, we created a climate high-risk window that we consider represents a high risk for the spread of H7N9 (temperature 4–14 °C and RHU 65–90%) and encompassed 192 H7N9 infections (55.01%), as well as a broader climate moderate-risk window that we consider represents a moderate risk for the spread of H7N9 (temperature 0–22  °C and RHU 40–95%), encompassing 334 infections (95.70%). According to these criteria, weeks 41–43 in North China, 3–14 and 46–53 in Central China, and 1–8 and 49–53 in South China commonly satisfy the criteria of the climate high-risk window. Furthermore, in weeks 8–23 and 35–48 in North China, 1–21 and 39–53 in Central China, and 1–17 and 43–53 in South China, extensive surveillance should be performed (Fig. 3A).

For H5N1 virus infection, the high-risk window corresponded to a temperature interval of 4–18 °C and pressure interval of 980–1025 kPa, with the moderate-risk window of 2–22 °C and 980–1025 kPa (Fig. 2D). As shown in Fig. 3B, week 44 in North China, 3–7, 11–14, and 46–52 in Central China, and 2–3, 7–8, and 47–51 in South China commonly satisfy the criteria of the climate high-risk window.

Furthermore, based on the mean temperature-humidity and temperature-atmospheric pressure during 2003–2013, the predictions of city distribution and month distribution were calculated (Supplementary Fig. S1, Table S1 and S2). We found that the occurrence of H5N1 influenza virus was concentrated in March, April, October, and November, while H7N9 influenza peaked in January and February. Darker color in the figure corresponds to a higher occurrence ratio of influenza in a specific province.

To determine whether the risky windows were statistically meaningful, we used the T-test to examine the differences in case numbers within and outside risky windows. For each province *i*, we counted the cases that met the criteria of the risky windows (*i.e*., *N*_*r,i*_) and did not meet these criteria (*i.e*., *N*_*n,i*_), respectively. Then, T-tests were performed to examine the mean value difference between *N*_*r,i*_ and *N*_*n,i*_ across all provinces. The null hypothesis is that the means value of *N*_*r,i*_ and *N*_*n,i*_ are the same, indicating that the risky window is not statistically meaningful. The alternate hypothesis is that the mean values are different, suggesting that the risky window is statistically significant. The T-test was performed on both H7N9 and H5N1, yielding *p*-values of 0.04024 and 0.03925, respectively. This suggests that case numbers within and outside risky windows are significantly different for both H7N9 and H5N1.

## Discussion

Both avian H5N1 and the newly emerged H7N9 influenza viruses are highly pathogenic AIV subtypes that can directly infect humans and cause severe diseases with high mortality. Unlike seasonal influenza, human infection with these two subtypes is sporadic, and little is known about the factors underlying the occurrence of human cases and the environmental factors favoring the occurrence of human infections. Herein, we identified that temperature, humidity, and atmospheric pressure are potential warning meteorological factors for the incidence of both H5N1 and H7N9 subtype infections.

The dependence of influenza virus transmission on environmental factors, including temperature, humidity, and atmospheric pressure, has been documented by many previous studies^18^,^19^,^20^,^21^. However, we found that the most adaptive meteorological conditions for the occurrence of the human cases of these two AIV subtypes are different. For example, most H5N1 cases appeared when the atmospheric humidity was 60–70%, whereas H7N9 cases predominantly occurred when the humidity was between 70–80%. In addition, differential atmosphere pressure and wind speed favor the occurrence of H7N9 and H5N1 cases, respectively. However, what underlies these differences is unknown and deserves further investigation. Several lines of evidence indicate that the high incidence of the featured seasonal pattern of H5N1 virus infection in poultry in China^22^,^23^, consistent with the human cases of H5N1 influenza virus in our study. Furthering the understanding of the correlation between the survival ability of the viruses relative to climatic factors should be considered.

Aside from meteorological factors, many other factors also impact the occurrence of human cases of H5N1 and H7N9 infection^24^,^25^. The migration of wild birds and live poultry transactions play an important role in spreading H5N1 and H7N9 viruses. The occurrence of human H5N1 cases has been suggested to overlap with wild bird flyways^26^. Although it is also reported that H7N9 virus is found only in chickens, ducks, and pigeons at live poultry markets and that no migratory birds have tested positive for the virus, migratory birds are implicated in the transmission of the virus. The regions with high incidences of H5N1 and H7N9 are places located along migratory bird flyways and also have surrounding poultry farms serving the densely populated areas that have many live poultry markets^27^.

To assess the different roles that various climatic factors play in H7N9 and H5N1 infections, we applied PCA to patient-specific meteorological data, and the results suggest that the first two principle components (PC1 and PC2) can be used to estimate correlations. Temperature factors and relative humidity are the dominant variables for PC1 and PC2 (respectively) for H7N9, while temperature and pressure are the key factors for H5N1 cases. These results suggest the possibility of using temperature and relative humidity to approximate the effects that the climate factors have upon human H7N9 infection. Just as compared with highly pathogenic AIV (HPAIV), the H5N1 virus shows differential environmental factors. Although climatic factors exhibit a strong correlation with H7N9 or H5N1 infection, other crucial factors doubtlessly include the density of the pathogen virus, human behavior, and population density.

Several previous studies depict risk maps in terms of H5N1/H7N9 infection in birds^7^,^21^,^28^,^29^,^30^. There are similar spatial distributions with H7N9 infections in humans but differential spatial distribution with H5N1 infections in humans. The risk areas for human H5N1 infection were more concentrated in South and Central China in our analysis. HPAIV viruses are generally thought to arise in poultry after domestic birds become infected by LPAI H5 and H7 viruses from the wild-bird reservoir^31^. The probable long co-existence of multiple viruses in multiple hosts provides the driving force for the virus to adapt to infect humans. Furthermore, it is difficult to identify humans, birds, or pigs infected with subclinical H7N9/H5N1 viruses from clinical signs, and it is extremely difficult to conduct large-scale surveillance to identify virus infections in humans, birds, or pigs for an extended period. Therefore, we must pay more attention to overlap areas.

The LPMs act as a melting pot for a variety of viruses at a range of densities, and market networks with numerous trade connections should permanently be closed down to avoid future avian influenza virus infections. However, there is vast number of small-scale poultry producers, supplying a strong demand for live poultry in China. Consequently, permanently closing LPMs would likely prove unpopular, and therefore, their periodic closure may represent a more viable compromise. The climate risk windows delineated in this study should be considered as important references for the guidance of any such solution. This strategy of metrological factor-based prediction can avoid large-scale live poultry culling and blindly closing live poultry markets to save a large amount of social resources.

## Materials and Methods

### Outcome Data

We collected the H5N1 and H7N9 influenza data reported on the Chinese mainland from the World Health Organization (WHO)’s Global Influenza Program (GIP, http://who.int/influenza/). The datasets used in this research included all human infection events of H5N1 and H7N9 on the Chinese mainland. The H5N1 dataset included 47 records from the first case reported on November 25, 2003 to the latest case reported on December 27, 2013. Similarly, the H7N9 dataset contained all 350 records from February 19, 2013 to March 4, 2014 in China (129 from 2013 and 221 from 2014). Both datasets included the basic information (reported province and reported date) for each patient.

The climatic data were collected from approximately 827 ground-based weather stations in the study area for the period from January 2003 through December 2013. The China Meteorological Data Sharing Service System (CMDSSS, http://data.cma.cn) stations across China with standard equipment for monitoring meteorological variables. The CMDSS data are widely used in climatic research, planning, and prediction analysis in China^30^,^32^. The climate dataset contained the air temperature, ground surface temperature, relative humidity, wind speed, and atmospheric pressure of all meteorological stations in the reported provinces at the reported dates for each record. There is more than one meteorological station in each province, and the site-level climate datasets were aggregated into the province-level (average for the climate factors), which are able to represent the weather situation in the study areas. Furthermore, several indices were derived from the province-level climate data for further analysis, *i.e*., the maximum, minimum, and mean value of the air temperature (TEM.mean, TEM.high, and TEM.low), the minimum and mean value of the relative humidity (RHU.mean and RHU.low), the mean value of the ground surface temperature (GST.mean), the pressure (PRS.mean), and the wind speed (WIN.mean) on each reported date. Briefly, the average daily data from each weather station in the province, observed four times on a daily basis (00:00, 06:00, 12:00, and 18:00), were used after removing outliers. According to province-level climate data, the daily mean data were selected to average total weather stations of each province from January 2003 through December 2013. The monthly mean data were calculated from the daily reports of each province.

### Statistical Analyses

#### Exploratory Data Analysis (EDA)

First, we employed EDA to summarize the data distribution and statistical characteristics of our datasets for both H5N1 and H7N9 infection cases. We analyzed the distribution of all potential meteorological factors for the H5N1 and H7N9 datasets using histograms, respectively.

#### Two-sample T-test

To further determine whether the climatic factors differ between the H5N1 events and H7N9 events, a series of two-sample T-tests were employed in our research due to the unequal sample sizes of the two groups. All hypothesis tests had a similar null hypothesis that the mean values of specific factors (X) are equal. Then, a T statistic was obtained by the equation below,


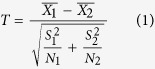


where 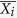, 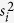, and *N*_i_ represent the mean value, variance, and sample size of a specific factor of the *i*th group (*i.e.*, Group 1: H5N1; Group 2: H7N9), respectively. Statistically, this statistic denotes a T distribution with the degree of freedom of df, which was calculated as below,


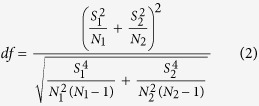


Given the significance level of a *p*-value (*i.e.*, 0.05 in this study), the null hypothesis should be rejected, which indicates that the mean values of the specific factors differ between the H5N1 events and H7N9 events, if the calculated T statistic meets the following critical region,


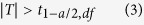


where *t*_1−*a*/2,*df*_ is the critical value of the *t* distribution with the degree of freedom of *df*.

#### Pearson’s Chi-squared Test

In addition to the T-tests, Pearson’s Chi-squared tests were used to examine the dependence of H5N1 events and H7N9 events on the occurring month and climatic factors. The null hypothesis of the Pearson’s Chi-squared test is that a specific factor is statistically independent from the occurrence of H5N1 and H7N9. Considering that the Pearson’s Chi-squared test could only be applied to the categorical variables, all continuous variables, including age, and all climate factors were converted into categorical variables by dividing observations into several groups using pre-defined thresholds.

Specifically, all observations of H5N1 and H7N9 were divided into: 1) three groups for occurring month, (Group 1: January to March; Group 2: April to June; and Group 3: July to December); 2) nine groups for air temperature, from –15 to 30 °C with a step of 5 °C; 3) eight groups for relative humidity, from 20% to 100% with a step of 10%; and 4) eight groups for ground surface temperature, from –10 to 30 °C with a step of 5 °C, separately. After the grouping procedure, the counts of all observations in each group could be exhibited as in the Table 5,

Accordingly, a *χ*^2^ statistic was calculated based on the following equations,


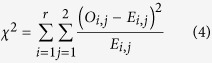



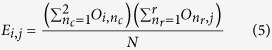


where *E*_i,j_ is the expectation value of each group at the *i*th row and *j*th column, and *N* is the total sample size of these two datasets. Statistically, this calculated *χ*^2^ statistic denotes a Chi-squared distribution with the degree of freedom (*df*) equal to *r*-1. Therefore, if the calculated *χ*^2^ meets the following critical region, we rejected the null hypothesis, which indicates that this factor is dependent on the occurrence of H5N1 and H7N9,


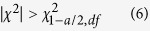


where *χ*^2^_1-*a*/2,*df*_ is the critical value of the Chi-squared distribution, *a* is the significance level (*i.e*., 0.05 in this study), and *df* is the degree of freedom.

#### PCA

In addition to the descriptive analysis applied for our datasets, we also deployed PCA for both datasets to determine relative contributions of each meteorological variable for both H7N9 and H5N1 cases and to reduce the dimensions of meteorological factors for further analysis. This quantitative analysis yielded better insight into the correlation between H7N9/H5N1 infection cases with dominant meteorological factors.

## Additional Information

**How to cite this article**: Li, J. *et al.* Identification of climate factors related to human infection with avian influenza A H7N9 and H5N1 viruses in China. *Sci. Rep.* **5**, 18094; doi: 10.1038/srep18094 (2015).

## Supplementary Material

Supplementary video 1

Supplementary video 2

Supplementary Materials

## Figures and Tables

**Table 1:** Geographical distribution of H5N1 and H7N9 influenza infections.

	**Province**	H5N1*	**H7N9**#
Cases of human infection with H5N1 and H7N9	Guizhou	4	1
	Liaoning	1	0
	Xinjiang	3	0
	Jinlin	0	1
	Shanxi	1	0
	Shandong	1	2
	Hebei	0	1
	Henan	0	3
	Beijing	2	4
	Hubei	2	0
	Hunan	8	17
	Sichuan	3	0
	Jiangxi	1	5
	Anhui	5	8
	Jiangsu	2	39
	Shanghai	1	33
	Zhejiang	1	127
	Fujian	4	20
	Guangdong	5	84
	Guangxi	3	5

^*^From November 25, 2003 to December 27, 2013.

^#^From February 19, 2013 to March 4, 2014.

**Table 2:** Null hypothesis 1: Mean values of this factor for H5N1 and H7N9 infection are the same.

**Factor**	***p*****-value**	**Result**	**Meaning**
TEM.mean	0.8444	Accept null hypo	Mean value is not different
RHU.mean	0.02039	Reject null hypo	Mean value is different
GST.mean	0.9303	Accept null hypo	Mean value is not different
PRS.mean	8.004e-05	Reject null hypo	Mean value is different
WIN.mean	1.304e-06	Reject null hypo	Mean value is different

**Table 3:** Null hypothesis 2: H5N1/H7N9 infection is independent from this factor.

**Factor**	***p*****-value**	**Result**	**Meaning**
TEM.mean	1.602e-06	Reject null hypo	H5/H7 is dependent on this factor
RHU.mean	0.008191	Reject null hypo	H5/H7 is dependent on this factor
GST.mean	2.348e-06	Reject null hypo	H5/H7 is dependent on this factor
PRS.mean	2.2e-16	Reject null hypo	H5/H7 is dependent on this factor
WIN.mean	2.958e-06	Reject null hypo	H5/H7 is dependent on this factor

**Table 4:** Comparison of first three principle components between H7N9 and H5N1 influenza infections.

	**H7N9**			**H5N1**		
	**Comp.1**	**Comp.2**	**Comp.3**	**Comp.1**	**Comp.2**	**Comp.3**
Standard deviation	1.979	1.389	1.085	1.938	1.190	1.137
Proportion of Variance	49.11%	24.20%	14.77%	46.92%	17.69%	16.17%
Cumulative Proportion	49.11%	73.31%	88.08%	46.92%	64.61%	80.78%
Loading:						
TEM.mean	**−0.498**	0.003	**−**0.089	**−0.509**	0.043	0.124
TEM.high	**−0.487**	0.041	0.021	**−0.453**	0.364	0.122
TEM.low	**−0.444**	**−**0.116	**−**0.302	**−0.477**	**−**0.261	0.008
RHU.mean	0.012	**−0.691**	**−**0.052	**−**0.182	**−**0.289	**−**0.670
RHU.low	0.057	**−0.675**	**−**0.188	0.001	**−**0.005	**−**0.108
WIN.mean	0.064	0.149	**−**0.775	0.093	**−**0.336	0.703
PRS.mean	0.256	0.157	**−**0.510	**−**0.119	**−0.769**	0.023
GST.mean	**−0.495**	0.066	**−**0.046	**−0.503**	0.101	0.123

**Table 5:** Contingency table for the Pearson’s Chi-squared independence test.

	**H5N1**	**H7N9**
Group*1*	*O*_1,1_	*O*_1,2_
…	…	…
Group *r*	*O*_r,1_	*O*_r,2_

*r* is the number of the group, and *O*_r,1_ and *O*_r,2_ represent the number of observations in the *r*th group of H5N1 and H7N9, respectively.
