# Supplementary material for: Identification of climate factors related to human infection with avian influenza A H7N9 and H5N1 viruses in China
Source: Sci Rep. 2015 Dec 11;5:18094. doi: 10.1038/srep18094 (PMC4676028; doi:10.1038/srep18094)
Supplement: Supplementary Materials [file srep18094-s3.pdf]

Identification of climate factors related to human infection with avian influenza A H7N9 and H5N1 viruses in China

Jing Li[1], Yuhan Rao[2,3], Qinglan Sun[4], Xiaoxu Wu[5], Jiao Jin[6], Yuhai Bi[1], Jin Chen[5], Fumin Lei[7], Qiyong Liu[8], Ziyuan Duan[9], Juncai Ma[4], George F. Gao[1], Di Liu[1,4] and Wenjun Liu[1*]

[1]CAS Key Laboratory of Pathogenic Microbiology and Immunology, Institute of Microbiology, Chinese Academy of Sciences, Beijing, 100101, China

[2]Department of Geographical Sciences, University of Maryland, College Park, Maryland, 20740, USA

[3]State Key Laboratory of Earth Surface Process and Resources Ecology, Beijing Normal University, Beijing, 100875, China

[4]Network Information Center, Institute of Microbiology, Chinese Academy of Sciences, Beijing, 100101, China

[5]College of Global Change and Earth System Sciences, Beijing Normal University, Beijing, 100875, China

[6]School of Mathematical Sciences, Beijing Normal University, Beijing, 100875, China

[7]CAS Key Laboratory of Zoological Systematics and Evolution, Institute of Zoology, Chinese Academy of Sciences, Beijing, 100101, China

[8]State Key Laboratory for Infectious Disease Prevention and Control, National Institute for Communicable Disease Control and Prevention, Chinese Center for Disease Control and Prevention,

Beijing, 102206, China

[9]Institute of Genetics and Developmental Biology, Chinese Academy of Sciences, Beijing 100101, China

***Corresponding author:**

Wenjun Liu

CAS Key Laboratory of Pathogenic Microbiology and Immunology, Institute of Microbiology, Chinese Academy of Sciences, Beijing, 100101, China

E-mail: liuwj@im.ac.cn

Supplement table 1. Predictions of the city and month distribution of H5N1 influenza infections[a].

Province	Jan[b]	Feb	Mar	Apr	May	Jun	Jul	Aug	Sep	Oct	Nov	Dec	Total days[c]
Jiangsu	100	181	281	309	238	0	0	0	108	276	220	131	1844
Guangdong	341	298	300	149	0	0	0	0	0	0	230	310	1628
Hebei	0	0	175	126	0	0	0	0	169	270	155	0	895
Henan	91	212	329	308	201	0	0	0	218	310	286	187	2142
Shanghai	110	183	267	298	215	53	0	0	0	238	217	137	1718
Fujian	340	305	335	261	97	0	0	0	0	198	284	310	2130
Tianjin	0	70	239	317	227	53	0	0	206	265	167	0	1544
Beijing	0	0	259	314	229	71	0	0	275	310	193	0	1651
Hubei	252	275	335	297	169	0	0	0	122	295	298	286	2329
Hunan	273	280	332	273	155	0	0	0	99	273	294	292	2271
Chongqing	196	87	64	0	0	0	0	0	0	71	116	186	720
Liaoning	0	0	158	325	336	203	0	67	311	304	139	0	1843
Shandong	0	137	294	326	294	79	0	0	224	310	284	113	2061
Jilin	0	0	0	58	0	0	0	0	131	175	0	0	364
Guangxi	341	291	296	146	0	0	0	0	0	69	240	310	1693
Zhejiang	282	290	339	317	215	0	0	0	0	249	295	287	2274
Jiangxi	285	301	335	283	131	0	0	0	65	245	296	299	2240
Anhui	183	248	333	309	200	0	0	0	115	296	297	237	2218

a, Predictions of the city distribution and month distribution were calculated according to the moderate risk window (temperature = 4-18 ℃ and pressure = 980-1025 kPa).

b, Days of risk in each month of each province during 2003-2013.

c, Total days of risk in each month or each province during 2003-2013

Supplement table 2. Predictions of the city and month distribution of H7N9 influenza infections[a].

Province	Jan[b]	Feb	Mar	Apr	May	Jun	Jul	Aug	Sep	Oct	Nov	Dec	Total days[c]
Guizhou	172	192	200	79	0	0	0	0	0	0	199	260	1102
Guangdong	128	83	0	0	0	0	0	0	0	0	0	0	211
Hebei	0	0	0	0	0	0	0	0	0	77	0	0	77
Beijing	0	0	0	0	0	0	0	0	0	86	0	0	86
Hubei	126	173	169	52	0	0	0	0	0	0	199	183	902
Chongqing	298	277	183	0	0	0	0	0	0	0	171	300	1229
Yunan	220	70	0	0	0	0	0	0	0	0	188	274	752
Liaoning	0	0	0	66	0	0	0	0	0	90	56	0	212
Sichuan	0	0	64	66	0	0	0	0	0	239	209	0	578
Shanxi	0	0	0	0	0	0	0	0	0	122	0	0	122
Jiangxi	188	181	161	0	0	0	0	0	0	0	122	194	846
Anhui	70	138	160	0	0	0	0	0	0		159	125	652
Jiangsu	68	137	156	67	0	0	0	0	0	0	158	133	719
Fujian	260	188	128	0	0	0	0	0	0	0		195	771
Shanghai	160	185	168	62	0	0	0	0	0	0	80	175	830
Henan	0	70	83	0	0	0	0	0	0	0	131	0	284
Tianjin	0	0	0	0	0	0	0	0	0	58	80	0	138
Hunan	163	180	176	52	0	0	0	0	0	17	153	206	947
Shaanxi	0	0	50	0	0	0	0	0	0	200	129	0	379
Shandong	0	0	58	66	0	0	0	0	0	0	100	0	224
Jilin	0	0	0	0	51	0	0	0	0	85	0	0	136
Guangxi	190	112	66	0	0	0	0	0	0	0	0	84	452
Zhejiang	193	209	171	50	0	0	0	0	0	0	101	184	908

a, Predictions of the city distribution and month distribution were calculated according to the high risk window (temperature = 4-14 ℃ and RHU = 65-90%).

b, Days of risk in each month of each province during 2003-2013.

c, Total days of risk in each month or each province during 2003-2013

Figure S1

A

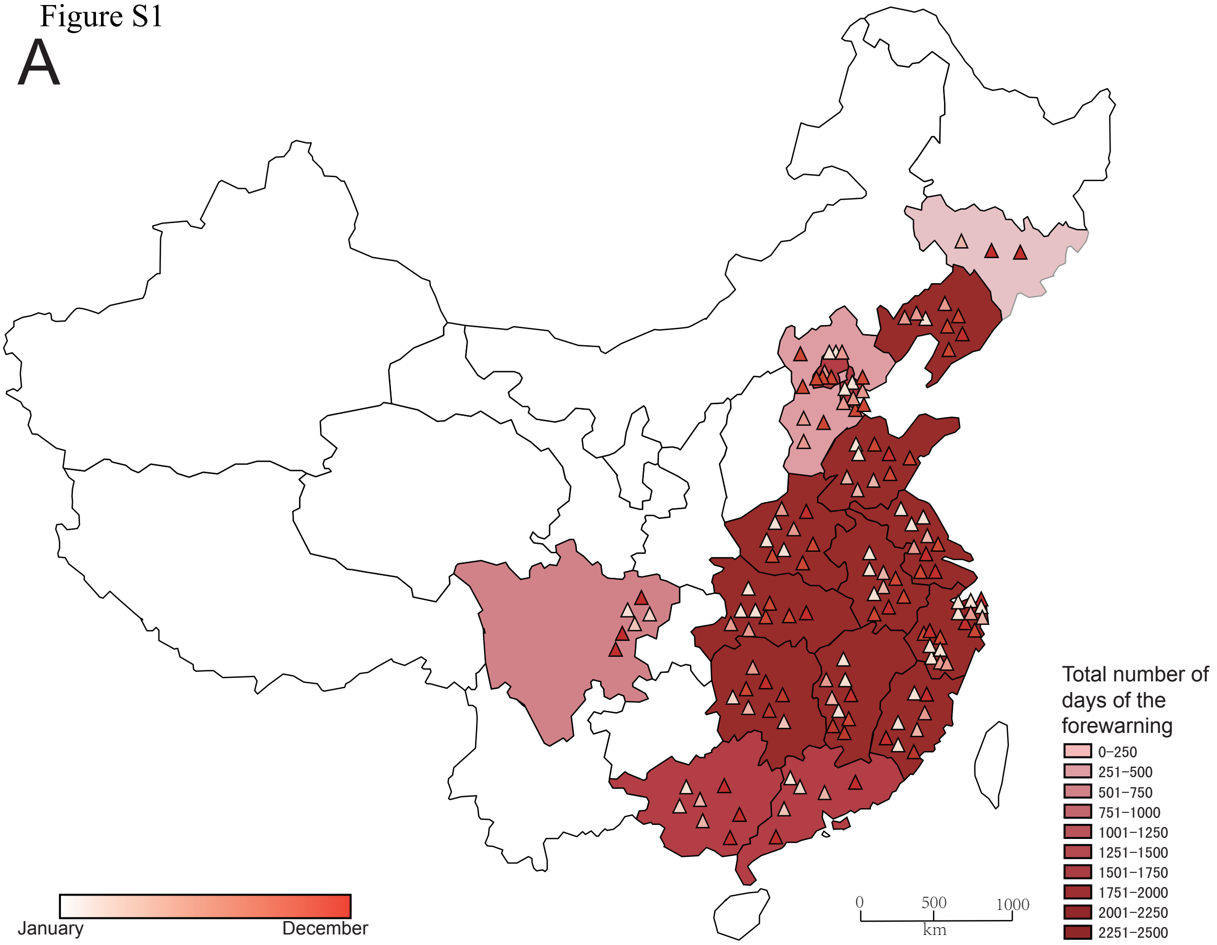

Total number of days of the forewarning
0-250
251-500
501-750
751-1000
1001-1250
1251-1500
1501-1750
1751-2000
2001-2250
2251-2500
0
500
1000
km
January
December

B

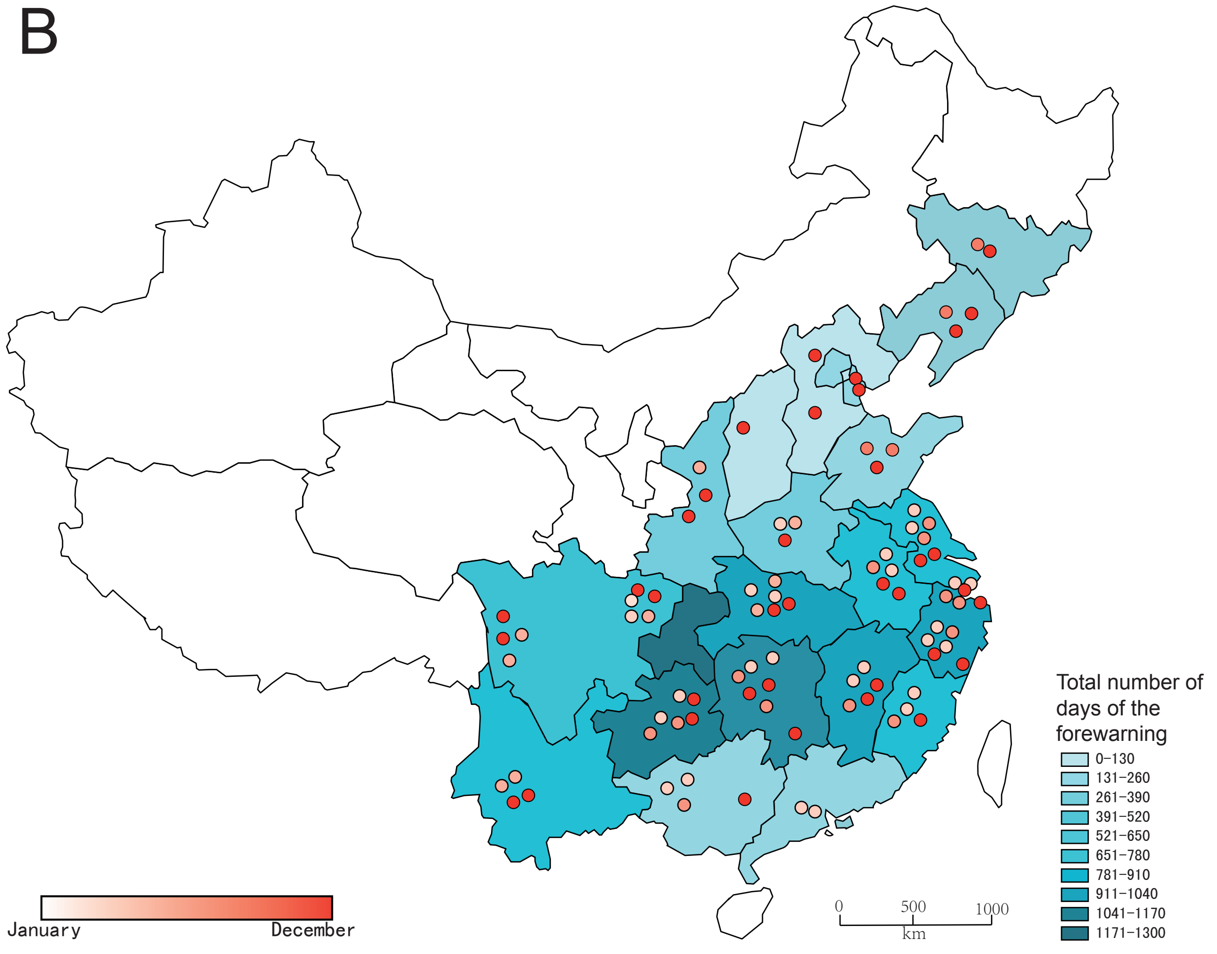

Total number of days of the forewarning
0-130
131-260
261-390
391-520
521-650
651-780
781-910
911-1040
1041-1170
1171-1300
0
500
1000
km
January
December

Figure Legends

Figure S1: A map displaying the predictions of city distribution and month distribution indicating the probability of an influenza peak, based on the mean temperature-humidity and temperature-atmosphere pressure during 2003-2013. (A) H5N1 influenza infection (B) H7N9 influenza infection. Provinces are shaded according to total days of the forewarning for H7N9 and H5N1 infection. The calendar dates of forewarning illness are represented by symbols.

Video S1: 3D scatter plot animations for H5N1 generated using MATLAB R2015a.

Video S2: 3D scatter plot animations for H7N9 generated using MATLAB R2015a.
